# The impact of hydrogen sulfide on gut microbiota of diabetic mice with lower limb arterial ischemia

**DOI:** 10.1186/s12866-026-05167-5

**Published:** 2026-06-05

**Authors:** Xuan Qiu, Wenxiao Li, Man Zhang, Shibo Lei, Hao Chen, Xiaohan Wang, Yuxin Miao, Zheng Yu, Yuming Wu, Zhiyong Hou

**Affiliations:** 1 Hebei Medical University Clinical Medicine Postdoctoral Research Station, Shijiazhuang, Hebei China; 2 Department of Endocrinology, Third Hospital of Hebei Medical University, Shijiazhuang, Hebei China; 3 Department of Orthopaedic Surgery, Third Hospital of Hebei Medical University, Shijiazhuang, Hebei China; 4 Human Microbiome and Health Group, Department of Microbiology, School of Basic Medical Science, Central South University, Changsha, Hunan China; 5 Department of Physiology, Institute of Basic Medicine, Hebei Medical University, Shijiazhuang, Hebei China; 6 The Key Laboratory of Neural and Vascular Biology, Ministry of Education, Shijiazhuang, Hebei China; 7 Hebei Key Laboratory of Cardiovascular Homeostasis and Aging, Shijiazhuang, Hebei China; 8 Engineering Research Center of Orthopedic Minimally Invasive Intelligent Equipment, Ministry of Education, Shijiazhuang, Hebei China; 9 Key Laboratory of Biomechanics of Hebei Province, Shijiazhuang, Hebei China; 10 NHC Key Laboratory of Intelligent Orthopaedic Equipment, Shijiazhuang, Hebei China

**Keywords:** Diabetes, Hindlimb ischemia, Hydrogen sulfide, Gut microbiota

## Abstract

**Background:**

The prevalence of hindlimb ischemia (HLI) associated with diabetes mellitus (DM) is high. However, its prevention and treatment face significant challenges. This study explored the effects of hydrogen sulfide (H_2_S) intervention in mice with DM and HLI, while concurrently investigating its regulatory effects on gut microbial homeostasis.

**Methods:**

The diabetic model in C57BL/6J mice was established through intraperitoneal injection of streptozotocin. The HLI model was created by ligating and severing the femoral artery, with subsequent initiation of a 21-day exogenous H_2_S intervention. Fecal samples from the mice were collected at four time points: before model establishment, 3 days after successful induction of the diabetes model, 3 days after establishment of the HLI model, and after 21 days of H_2_S intervention for metagenomic analysis. Body weight, blood glucose levels, and hindlimb blood flow in the mice were monitored. Additionally, functional assessment and histopathological examination of the ischemic skeletal muscle were performed to evaluate contractile and morphological properties.

**Results:**

H_2_S administration significantly enhanced hindlimb blood perfusion and restored plasma H_2_S concentrations in diabetic mice with HLI, concurrently improving both function and morphological integrity of the ischemic skeletal muscle. Bacterial abundance at the phylum level showed changes over the course of the experiment, particularly in *Bacteroidetes* and *Firmicutes*. In the DM + HLI group, the *Firmicutes*-to-*Bacteroidetes* ratio was significantly elevated; however, H_2_S treatment downregulated this alteration. H_2_S intervention modulated the abundance of various bacterial species, increasing *Lactobacillus murinus* and *Faecalibacterium prausnitzii*, while simultaneously downregulating inflammation-related bacteria such as *Ruminococcus sp. JE7A12*. Microbial network analysis revealed that the DM + HLI and H_2_S groups had lower network complexity than the control group. Furthermore, functional metagenomic profiling identified 28 differentially expressed genes, which were annotated to 8 primary and 30 secondary KEGG pathways, with 6 genes specifically enriched in carbohydrate metabolism pathways.

**Conclusion:**

Exogenous H_2_S administration improved hindlimb blood perfusion, restored contractile function, and preserved morphological integrity of ischemic skeletal muscle in diabetic mice with HLI. Concurrently, H_2_S treatment altered the abundance of gut microbiota, improving microbial balance. Targeting the gut microbiota via H₂S suggests a potential translational avenue that warrants causal investigation for the treatment of diabetic limb ischemia. Further studies are warranted to establish causal relationships and elucidate the underlying mechanisms linking H_2_S, gut microbiota, and vascular recovery.

**Supplementary Information:**

The online version contains supplementary material available at 10.1186/s12866-026-05167-5.

## Background

Peripheral artery disease (PAD) is a prevalent and severe form of systemic atherosclerosis, posing a significant global public health challenge [1]. Current statistics indicate that PAD impacts the health of over 230 million individuals worldwide and increases the risk of coronary heart disease, stroke, and limb amputation among affected patients [1, 2]. Notably, diabetes is a significant risk factor for the development of PAD [2]. In the U.S., more than 50% of leg amputation surgeries are attributed to the combination of diabetes and PAD [3]. Between 2009 and 2017, the one-year and five-year amputation rates for patients with both diabetes and PAD in the U.S. were reported to be 1.5% and 3%, respectively [4]. The current treatment approach for PAD mainly focuses on managing risk factors, as well as utilizing interventional and surgical methods, since there are no specific medications or therapies available at this time [1, 5]. Therefore, it is crucial to explore new therapeutic avenues and targets to prevent and treat diabetes-related PAD.

Research has demonstrated a link between diabetes mellitus (DM) and gut microbiota, highlighting the feasibility, safety, and clinical effectiveness of probiotic treatments for diabetes and its associated complications [6]. In recent years, numerous studies have indicated that gut microbiota plays a crucial role in the onset and progression of diabetic atherosclerosis [7]. Specific gut microbiota, such as *Coprococcus2*, *Family XI*, genera *Lachnoclostridium* and *Lachnospiraceae UCG001*, have been found to have a causal relationship with PAD [8, 9]. Indole, tryptophan, indole-3-propionic acid, and indole-3-aldehyde, along with other microbial metabolites, are negatively correlated with the progression of PAD [10]. Modulating gut microbiota may represent an effective and innovative therapeutic strategy for diabetes associated with PAD.

Hydrogen sulfide (H_2_S) is the third established gasotransmitter, after nitric oxide (NO) and carbon monoxide (CO). H_2_S at physiological concentrations helps stabilize the intestinal mucus layer and prevent the disruption of microbial biofilms [11]. Importantly, studies have demonstrated that H_2_S donors can maintain microbial homeostasis in mice with intestinal barrier injury [12], highlighting the regulatory role of H_2_S on gut microbiota. H_2_S in the gut is produced by host cells, bacteria, or derived from the diet [11]. The stability of gut microbiota also influences the production and concentration balance of H_2_S. Sulfate-reducing bacteria in the gut produce H_2_S, which modulates systemic concentrations, thereby connecting intestinal homeostasis to broader metabolic processes [13, 14]. At physiological levels under gut microbiota homeostasis, H_2_S helps maintain intestinal barrier integrity and prevents systemic inflammation [14]. Furthermore, gut microbiota-derived H_2_S has been shown to exhibit antihypertensive properties [15]. However, gut dysbiosis can lead to excessive H_2_S production, which impairs the intestinal barrier, compromises mitochondrial function, increases oxidative stress, and exacerbates inflammation. Additionally, it aggravates neurodegenerative processes by promoting neuroinflammation, modulating oxidative stress and immune responses, and worsening glial cell dysfunction [14]. *Desulfovibrio*, a H_2_S-producing sulfate-reducing bacterium, is enriched in the gut of patients with metabolic syndrome. It inhibits the production of intestinal glucagon-like peptide-1 (GLP-1) in mice through excessive H_2_S generation [13]. Similarly, enrichment of *Desulfovibrio* in patients with metabolic dysfunction-associated steatotic liver disease (MASLD)/metabolic dysfunction-associated steatohepatitis (MASH) and cholelithiasis leads to increased H_2_S production, which plays a significant role in the pathogenesis of liver inflammation and cholelithiasis [16, 17]. In summary, H_2_S and the gut microbiota form a bidirectional regulatory axis: physiological concentrations of H_2_S help maintain microbial homeostasis and exert organ-protective effects, whereas dysbiosis-induced abnormal H_2_S metabolism contributes to various disease processes by disrupting barrier function and promoting inflammatory responses. This intricate interplay underscores the need to elucidate how H_2_S-mediated modulation of the gut microbiota confers protection against diabetic peripheral artery disease.

Given this bidirectional regulatory axis, the influence of the H_2_S-gut microbiota interaction on peripheral vascular health has garnered increasing attention. H_2_S plays a significant role in the regulation of vascular function. Our team has discovered that H_2_S could improve macrovascular complications in diabetic patients by regulating autophagy of vascular smooth muscle cells [18]. Evidence also suggested that the gut microbiota could improve atherosclerosis by producing H_2_S [19]. Simultaneously, low concentrations of exogenous or endogenous H_2_S could protect blood vessels by modulating the gut microbiota [11]. A more thorough investigation into how H_2_S regulates the microbiota to protect peripheral vessels in diabetes could reveal new therapeutic strategies.

This study employed animal experiments and metagenomic analysis to explore the effects of H_2_S intervention in diabetic mice with hindlimb ischemia (HLI), with a particular emphasis on alterations of gut microbiota. The aim was to investigate whether the protective effects of H_2_S on peripheral arteries are connected to gut microbiota regulation, potentially providing new insights for treating diabetes-related peripheral artery occlusion.

## Materials and methods

### Animals

24 eight-week-old C57BL/6J mice were obtained from the Beijing Weitonglihua Laboratory Animal Center and housed in a temperature-controlled environment (25 ± 1℃) with a 12-hour light/dark cycle. The mice were kept four per cage with access to ample food and water. All animal procedures complied with the US National Institutes of Health Guide for the Care and Use of Laboratory Animals and received approval from the Animal Ethics Committee of Hebei Medical University (IACUC-Hebmu-P-2025028).

### Grouping and treatment

After a two-week adaptive feeding period, the mice were randomly divided into three groups: control group (*n* = 8), diabetes with lower limb arterial ischemia (DM + HLI) group (*n* = 8), and DM + HLI+H_2_S group (H_2_S group) (*n* = 8). The DM + HLI group and the H_2_S group received intraperitoneal injections of streptozotocin (STZ; Sigma, St. Louis, MO, USA; 50 mg/kg in 0.1 mol/L citrate buffer, pH 4.5) for five consecutive days to induce diabetes [20]. In contrast, the control group received intraperitoneal injections of citrate buffer. On the eighth day, three days after the final STZ injection, fasting blood glucose (FBG) levels were measured. Blood samples were collected from the tail vein, and glucose levels were assessed using a glucometer (ACCU-CHEK, Roche, Switzerland). Mice with FBG levels ≥ 16.7 mmol/L were considered to have successfully developed diabetes and were included in subsequent studies. On the eleventh day, the mice in the DM + HLI and H_2_S groups underwent HLI surgery. On the fourteenth day, three days after surgery, mice in both the control and DM + HLI groups received daily intraperitoneal injections of saline, while those in the H_2_S group received daily intraperitoneal injections of sodium hydrosulfide (NaHS, Sigma, St. Louis, MO, USA; 100 µmol/kg/d) [21, 22]. This dose was selected based on previous studies demonstrating its efficacy in improving vascular function in diabetic models without adverse effects [21, 22], and is intended to restore physiological H_2_S levels that are deficient in diabetes. Of note, we did not measure H₂S concentration in the gut lumen or portal circulation; therefore, the actual concentration of H₂S reaching the gut microbiota remains unknown. The drug intervention lasted for 21 days. Cage-side observations indicated no episodes of diarrhea or increase of mortality in the H_2_S group, and the general behavior and physical appearance of the mice remained normal throughout the study.

### HLI surgery

In brief, diabetic mice were anesthetized with ketamine (80 mg/kg) and xylazine (50 mg/kg). Following fixation and shaving of the left hindlimb, a 5-mm incision was made at its proximal end. The femoral artery was ligated at both proximal and distal sites, and the segment between ligatures along with its small branches was resected. The skin was meticulously sutured with 4 − 0 surgical thread [23]. Successful induction of ischemia was confirmed postoperatively by laser Doppler perfusion imaging.

### Blood perfusion assessment

Limb blood flow was measured using a Laser Doppler Flowmetry system (PeriFlux 5000 LDPM, Perimed AB, Sweden). This procedure was performed according to the detailed protocol described previously [24, 25]. Before each measurement, mice were anesthetized with isoflurane (1%–2%), and hind limb fur was removed. The animals were then placed on a 37 °C heating plate for 10 min to minimize temperature fluctuations during imaging. Subsequently, mice were placed on a black scanning platform for simultaneous bilateral hindlimb dorsal blood flow acquisition. Perfusion images were analyzed using PIMSoft software (PeriCam PSI System). The blood perfusion ratio was calculated as the flow in the left (ischemic) limb relative to that in the right (non-ischemic) limb [23]. To confirm successful induction of the HLI model and to track recovery over time, perfusion assessments were performed immediately after surgery and repeated on days 7, 14, and 21 post-operation.

### Stool sample collection

**Fig. 1:**
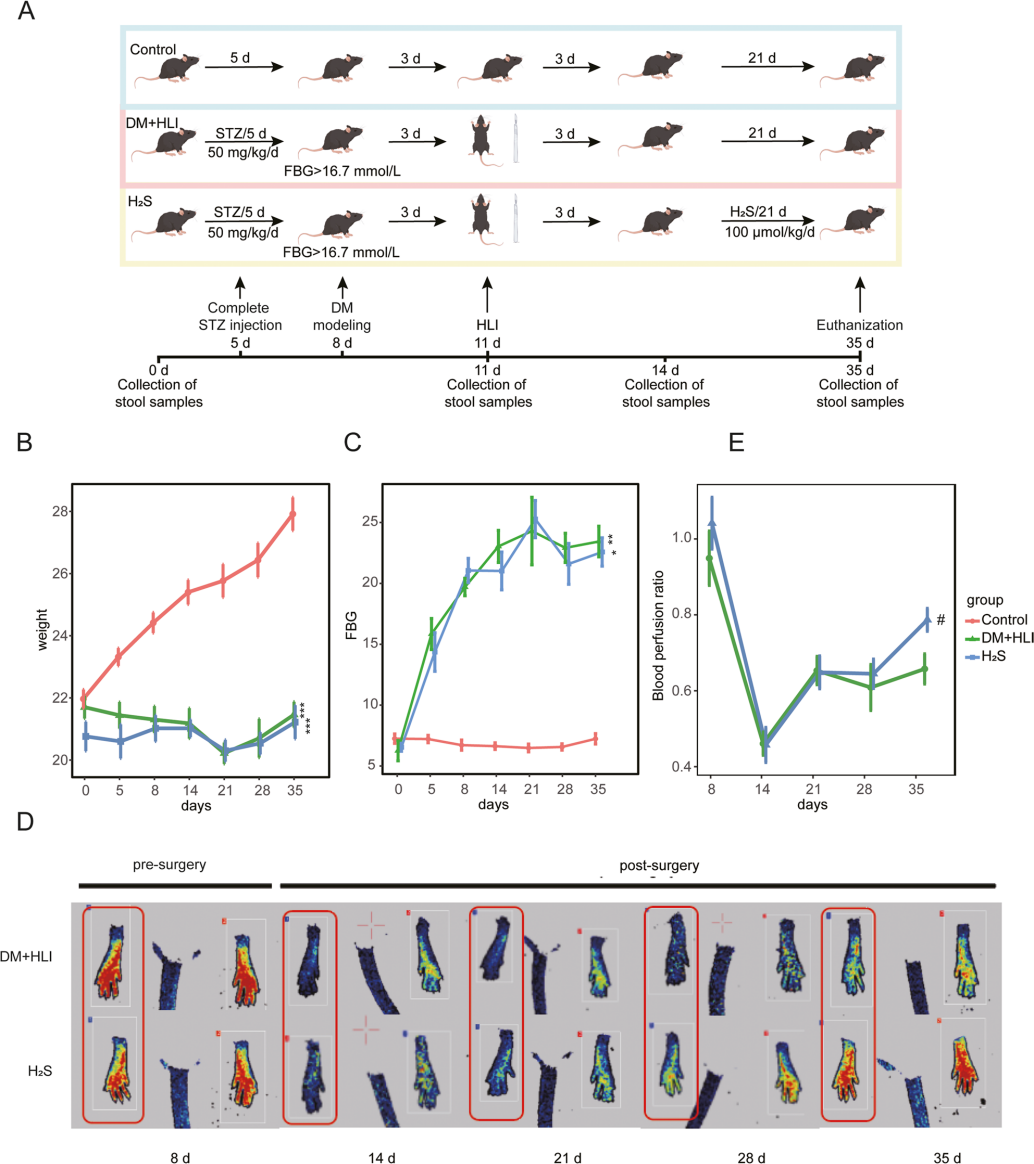
Experimental timeline and phenotypic characterization of mice. **A** Schematic diagram of the experimental design. **B** Body weight changes throughout the study period. **C** Dynamics of FBG levels. **D**, **E** Representative laser Doppler perfusion images of hindlimbs (**D**) and corresponding quantitative analysis of the perfusion ratio (ischemic/non-ischemic limb) (**E**). Data are presented as mean ± SEM. Statistical comparisons at the endpoint (day 35) were performed using one-way ANOVA with Bonferroni’s multiple comparison test for comparisons among three groups, or *t*-test for comparisons between two groups (*n* = 5; ^⁎^*P* < 0.05, ^⁎⁎^ *P* < 0.01, ^⁎⁎⁎^ *P* < 0.001 vs. control group; ^#^ *P* < 0.05 vs. DM + HLI group). DM, diabetes mellitus; HLI, hindlimb ischemia; H_2_S, Hydrogen Sulfide; FBG, fasting blood glucose; STZ, streptozotocin; SEM, standard error of the mean; ANOVA, analysis of variance

In this experiment, fecal samples were collected at four time points: before animal modeling (day 0), three days after successful diabetes modeling (day 11), three days post-HLI surgery (day 14), and on day 21 of drug intervention (day 35) (Fig. 1A). Prior to fecal collection, the mice were restrained, and their tails were lifted. Gentle pressure was applied to the lower abdomen to encourage defecation. Fresh feces were collected in sterile tubes and stored at -80 °C.

### Metagenome shotgun sequencing of the intestinal microbiome

Stool samples were randomly selected for metagenomic shotgun sequencing. Only the DM + HLI group at the third sampling time point had 4 samples; all other group-time point combinations (including the DM + HLI group at the other three time points) had 5 samples. Total genomic DNA was extracted from the stool samples using the OMEGA Soil DNA Kit (D5625-01) (Omega Bio-Tek, Norcross, GA, USA) according to the manufacturer’s protocol. The quantity and quality of the extracted DNA were subsequently assessed using a NanoDrop ND-1000 spectrophotometer (Thermo Fisher Scientific, Waltham, MA, USA) and agarose gel electrophoresis, respectively. Next, the Illumina TruSeq Nano DNA LT Library Preparation Kit was used to process the DNA and construct 400 bp insert libraries. Sequencing was performed on the Illumina HiSeq X-ten platform (Illumina, USA) using the PE150 strategy at Personal Biotechnology Co., Ltd. (Shanghai, China). The raw reads were then processed to obtain quality-filtered reads for subsequent analysis. Detailed bioinformatics methods are shown in supplementary materials.

### Hematological parameter assays

At the study endpoint (day 35, the day after the final NaHS injection), blood samples were obtained from overnight-fasted mice. After collection, samples were centrifuged at 3500 rpm for 10 min. The resulting plasma was stored at -80 °C for subsequent analysis. H_2_S levels were determined using a commercial colorimetric assay kit (E-BC-K355-M, Elabscience, Wuhan, China) according to the manufacturer’s instructions. Plasma concentrations of interleukin-6 (IL-6) and interleukin-1β (IL-1β) were measured using ELISA kits (IL-6: FY-EM5315; IL-1β: FY-EM1445; Feiyue Biotechnology Co., Ltd., Wuhan, China) following the protocols provided by the manufacturer.

### Muscle function testing

Mice were anesthetized using intraperitoneal injection of 20% urethane (4 ml/kg). The left gastrocnemius muscle was rapidly isolated and excised, then rinsed in oxygenated Krebs-Henseleit solution (composition in mM: NaCl (118.0), KCl (4.7), MgSO_4_·7H_2_O (1.2), CaCl_2_ (2.5), KH_2_PO_4_ (1.2), NaHCO_3_ (25.0), glucose (11.0), pH 7.4 ± 0.05) at 4 °C. The isolated gastrocnemius muscle was gently suspended vertically in a 10 ml bath filled with K-H solution (temperature: 37 ± 0.5 °C, pH 7.4 ± 0.05). The proximal end of the muscle was fixed, while the distal end was connected to a mechanical force transducer (AD Instruments, Australia). A preload of 1 g was applied to allow the skeletal muscle to acclimatize for 1 h, during which the bath solution was replaced every 15 min. Subsequently, the gastrocnemius muscle was stimulated via platinum electrodes using square-wave pulses (parameters: 5 V, 1 Hz, pulse width 1 ms) to obtain the maximum twitch force. Tetanic contraction force was measured using square-wave pulses (parameters: 5 V, 120 Hz, pulse width 1 ms, 30 cycles). Data acquisition and analysis were performed with LabChart 7 software (AD Instruments). Muscle contractile performance was evaluated based on the maximum rates of tension development and relaxation.

### Pathological observation

The left gastrocnemius muscle of mice was fixed in muscle mounting solution (G1111, Servicebio, Wuhan, China) and subsequently embedded in paraffin. Sections with a thickness of 5 μm were prepared and stained with hematoxylin and eosin (H&E) as well as Masson staining. Observations were conducted under an optical microscope at 40×objective magnification. Additionally, the left gastrocnemius muscle was rapidly dissected on ice and fixed in 4% glutaraldehyde solution. Ultrathin sections were prepared at the Electron Microscopy Laboratory of Hebei Medical University and observed under a transmission electron microscope (HT7800, Hitachi, Japan).

### Statistical analysis and visualization

Statistical analyses were performed using SPSS (version 26.0), prism (version 10.1.2) and R (version 4.4.0). The normality of the data was tested using the Shapiro-Wilk test. Differences for normally distributed data were analyzed using *t*-test or ANOVA with Bonferroni’s multiple comparison. Differences for non-normally distributed data was analyzed by Wilcoxon test, with Benjamini-Hochberg false discovery rate (FDR) adjustment applied specifically to pairwise comparisons among multiple groups. Effect sizes were quantified using the correlation coefficient *r*, calculated as *r* = *Z* / √*N*, where *Z* is the test statistic and *N* is the total sample size. Values of |*r*| < 0.1, 0.1–0.3, 0.3–0.5, and > 0.5 were considered negligible, small, medium, and large effects, respectively. The results were visualized using the ‘ggplot2’ package unless otherwise specified. Alpha diversity was assessed using the Shannon, Richness, Chao and Ace index, while β diversity was evaluated through Principal Coordinates Analysis (PCoA) based on Bray-Curtis distance. The “randomForest” package was used for random forest regression analysis. The interaction network between genera was visualized using the “igraph” package in the R and Gephi software (version 0.10.1). Differences in microbial composition were analyzed using Linear Discriminant Analysis Effect Size (LEfSe) and Differential Expression analysis of Sequencing data version 2 (DESeq2). A linear model was used to perform the regression analysis of the species. Non-metric multidimensional scaling (NMDS) was performed using the metaMDS function in the vegan package for ordination analysis. Differential gene expression analysis was conducted with DESeq2. Kyoto Encyclopedia of Genes and Genomes (KEGG) pathway enrichment analysis was performed using DAVID. Fisher’s exact test was applied with the whole genome background. *Spearman* correlation analysis with Benjamini-Hochberg FDR correction was used to evaluate associations between different microbial species and genes. A *p*-value of < 0.05 was considered statistically significant.

## Results

### The protective effect of H_2_S in diabetic mice with HLI

The experimental animal groupings and the time points for fecal sample collection are shown in Fig. 1A. Compared to the control group, the DM + HLI and H_2_S groups exhibited reduced weight gain (Fig. 1B). Following STZ treatment, FBG levels in both the DM + HLI and H_2_S groups increased significantly compared to the control group (Fig. 1C). HLI surgery obstructed blood flow to the left hind limb in mice in the DM + HLI and H_2_S groups (Fig. 1D, E). As time progressed after HLI surgery, the recovery of blood flow in the affected limb of the H_2_S group was superior to that in the DM + HLI group (Fig. 1D, E).

**Fig. 2:**
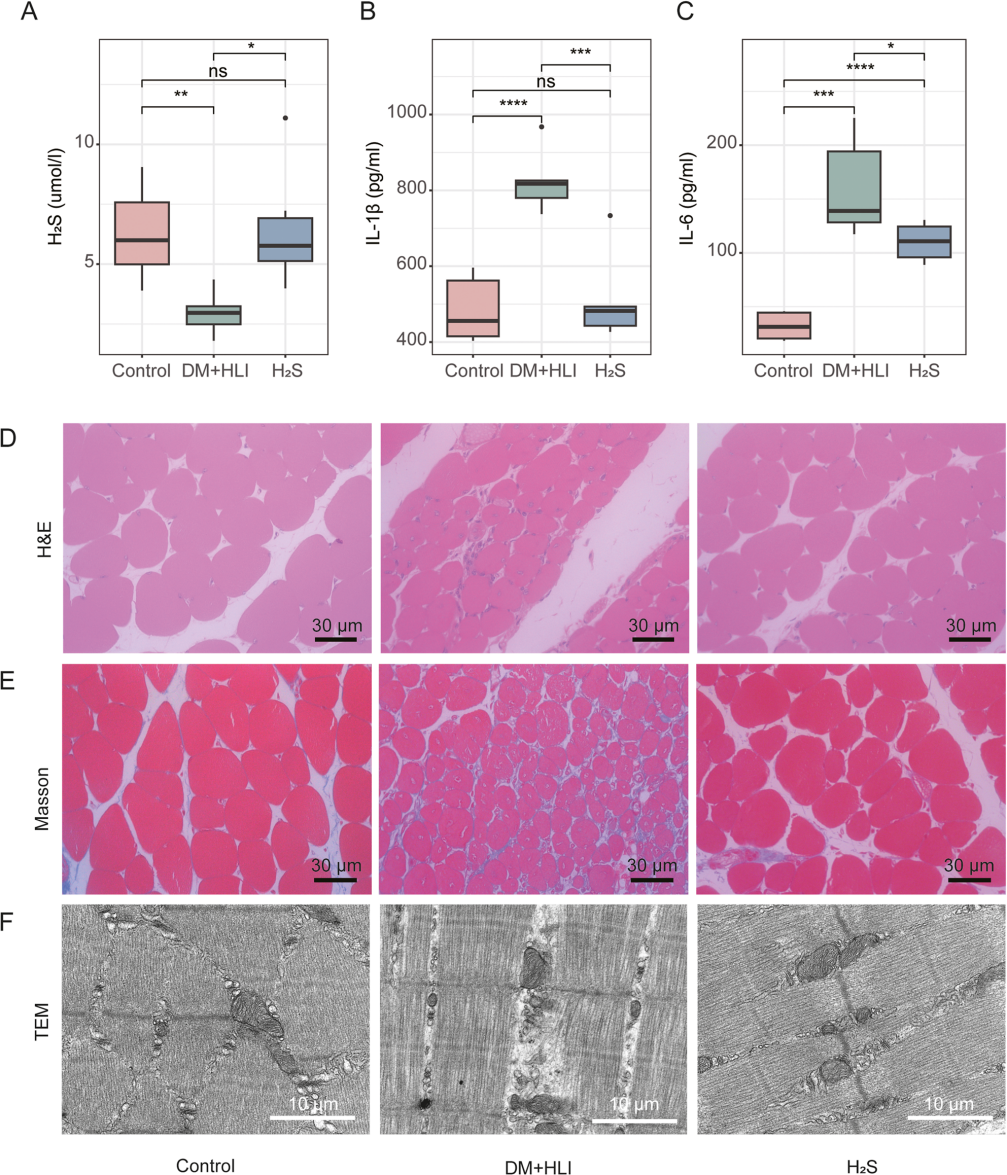
Comparative analysis of plasma biomarkers and skeletal muscle pathology among the three groups. **A**-**C** The quantitative analysis of H_2_S (**A**), IL-1β (**B**), and IL-6 (**C**) levels in plasma. **D**, **E** Representative micrographs of skeletal muscle sections stained with H&E (**D**) and Masson trichrome (**E**). Scale bar = 30 μm. **F** Representative TEM images of skeletal muscle. Scale bar = 10 μm. All data are presented as mean ± SEM (*n* = 6; ^ns^ *P* > 0.05, * *P* < 0.05, ^**^ *P* < 0.01, ^***^ *P* < 0.001, ^****^ *P* < 0.0001 by one-way ANOVA with Bonferroni’s multiple comparison test). DM, diabetes mellitus; HLI, hindlimb ischemia; H_2_S, Hydrogen Sulfide; IL-6, Interleukin-6; IL-1β, Interleukin-1β; H&E, hematoxylin-eosin; TEM, transmission electron microscopy; SEM, standard error of the mean; ANOVA, analysis of variance

As presented in Fig. 2A, the results showed that plasma H_2_S levels were reduced considerably in the DM + HLI model group compared to the control group. Exogenous H_2_S supplementation effectively reversed this reduction. Compared to the control group, the DM + HLI group showed significantly elevated levels of the inflammatory markers IL-6 and IL-1β, which were partially reversed by H_2_S treatment (Fig. 2B, C).

Morphological assessment of the ischemic gastrocnemius muscle was performed using H&E staining, Masson staining, and transmission electron microscopy (TEM). Optical microscopy observations revealed skeletal muscle cell swelling, sarcoplasmic dissolution, and neutrophil infiltration in the DM + HLI group (Fig. 2D), along with significant cellular atrophy and fibrosis (Fig. 2E). Notably, H_2_S intervention alleviated these changes (Fig. 2D, E). Electron microscopy observations showed neatly arranged myofibrils, clearly visible mitochondrial structures with relatively intact membranes, layered cristae within mitochondria, and intact nuclear morphology in the control group (Fig. 2F). In contrast, the DM + HLI group exhibited disrupted and broken myofibrils, blurred and dissolved mitochondria, and pyknotic nuclei (Fig. 2F). Thus, H_2_S intervention mitigated such damage (Fig. 2F).

Functionally, the DM + HLI group showed significantly impaired muscle contractility compared to controls, characterized by markedly reduced maximum contraction and relaxation rates (Fig. S1A-C). H_2_S treatment notably ameliorated these functional deficits (Fig. S1A-C).

### Dynamic changes in microbial diversity and species composition among the groups

**Fig. 3:**
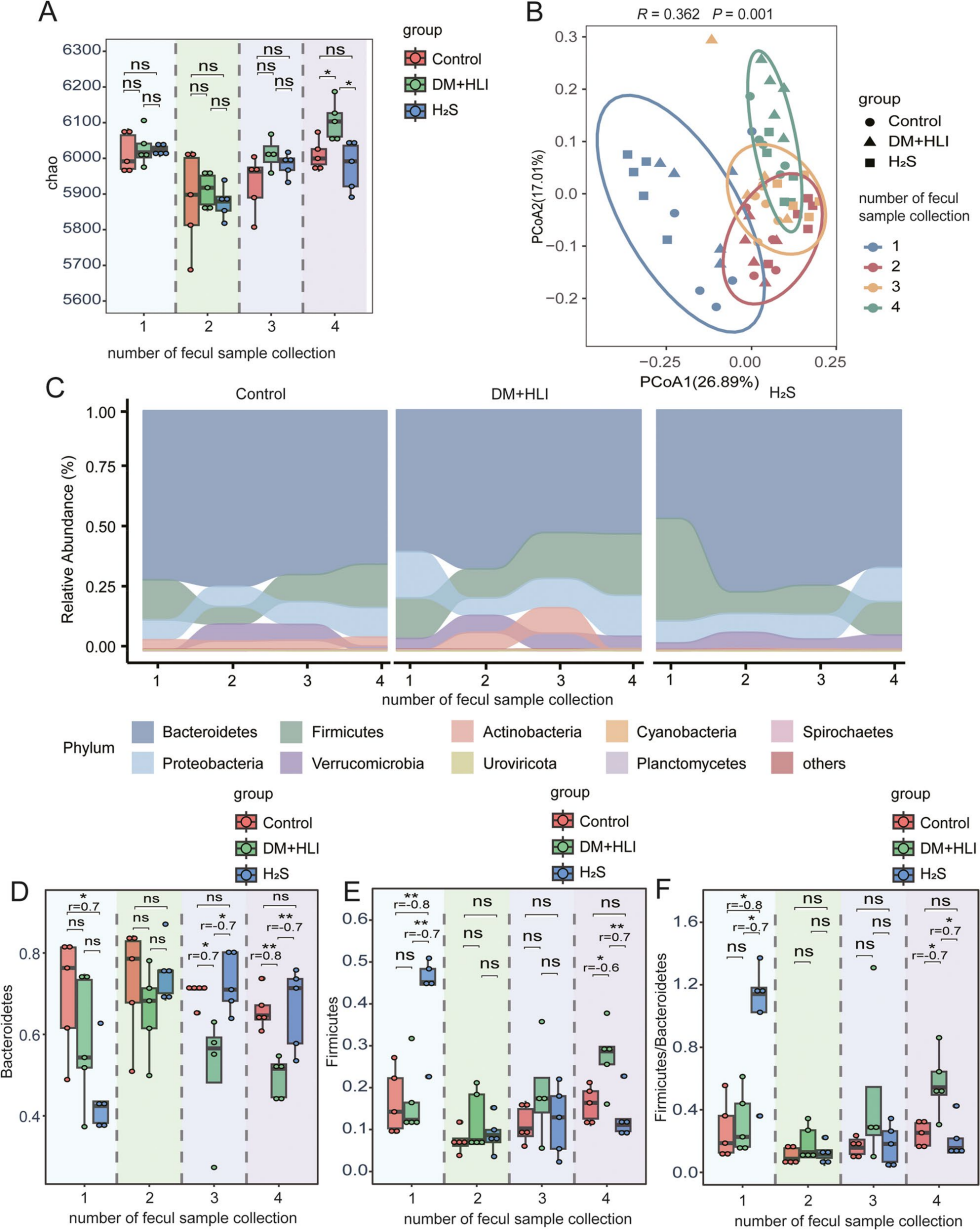
Temporal dynamics of gut microbial diversity and composition. **A** Comparative analysis of Chao index across the three groups at all four sampling time points. **B** PCoA analysis for the β diversity among the three groups. Each color represents a sampling time point, and each shape denotes an experimental group. **C** Temporal shifts in microbial community structure at the phylum level, presented as relative abundance. **D**-**F** Comparative analysis of *Bacteroidetes* abundance (**D**), *Firmicutes* abundance (**E**), and the *Firmicutes*-to-*Bacteroidetes* ratio (**F**) across the three groups at all four sampling time points. Data are presented as mean ± SEM. With the sole exception of the DM + HLI group at the third sampling time point (*n* = 4), all other groups at all time points had 5 samples (*n* = 5); ^ns^ *P* > 0.05, ^*^ *P* < 0.05, ^**^ *P* < 0.01 by Wilcoxon test with Benjamini-Hochberg FDR adjustment applied specifically to pairwise comparisons among multiple groups. Effect sizes were quantified using the correlation coefficient *r* (calculated as *r* = *Z* / √*N*, where *Z* is the test statistic and *N* is the total sample size). DM, diabetes mellitus; HLI, hindlimb ischemia; H_2_S, Hydrogen Sulfide; PCoA, principal coordinates analysis; SEM, standard error of the mean; FDR, false discovery rate

The mice were randomly allocated to groups after the acclimatization period to minimize cage effects. Data analysis then assessed both changes from baseline and inter-group differences at comparable later time points. Analysis of fecal samples collected at the baseline of the experiment revealed no statistically significant differences in α-diversity indices—including Richness, Chao, and Ace indices—among the groups (Fig. S2A-C). The Chao index exhibited dynamic changes over the experimental timeline. While no intergroup differences were observed at the first three sampling points, the index in the DM + HLI group was elevated compared to both the control and H_2_S groups at the final time point (Fig. 3A). The PCoA analysis indicated that microbial community β-diversity changed over time across all three groups (Fig. 3B). The 10 most abundant phyla were selected to investigate changes in microbial composition. In each group, the bacterial abundance at the phylum level showed changes as the experiment progressed, particularly in the *Bacteroidetes* and *Firmicutes* phyla (Fig. 3C). Following the preparation of the diabetes and HLI models, the abundance of *Bacteroidetes* in the feces of mice in the DM + HLI group gradually decreased, while the abundance of *Firmicutes* increased (Fig. 3C). The application of H_2_S partially reversed these changes (Fig. 3C). At the first sampling point (baseline), differences in the abundance of *Bacteroidetes*, *Firmicutes*, and their ratio were observed among the three groups, likely due to the cage-housed environment. Importantly, these differences were absent at the second sampling (three days after STZ modeling), confirming the comparability of the groups for subsequent analyses. Notably, analyses of the fecal samples collected during the third and fourth collections revealed that the abundance of *Bacteroidetes* in both the H_2_S group and the control group was significantly higher than in the DM + HLI group (Fig. 3D). Furthermore, fecal test results from the fourth collection demonstrated that the abundance of *Firmicutes* in the DM + HLI group was significantly greater than in the other groups (Fig. 3E). As a result, the *Firmicutes*-to-*Bacteroidetes* ratio exhibited notable differences, with the DM + HLI group showing a higher ratio compared to control group, while the H_2_S group displayed a lower ratio than the DM + HLI group (Fig. 3F). For the above comparisons, the values of |*r*| exceeding 0.5 indicated large effect sizes (Fig. 3D-F). Moreover, the random forest regression analysis showed that the lowest value of cross-validation error curves was observed when 17 species were included in the model for four time points. From the top 250 species in terms of abundance, 14 species that contributed most significantly to intergroup differences were selected (Fig. S3).

### The effects of H_**2**_S intervention on the gut microbiota in diabetic mice with HLI

**Fig. 4:**
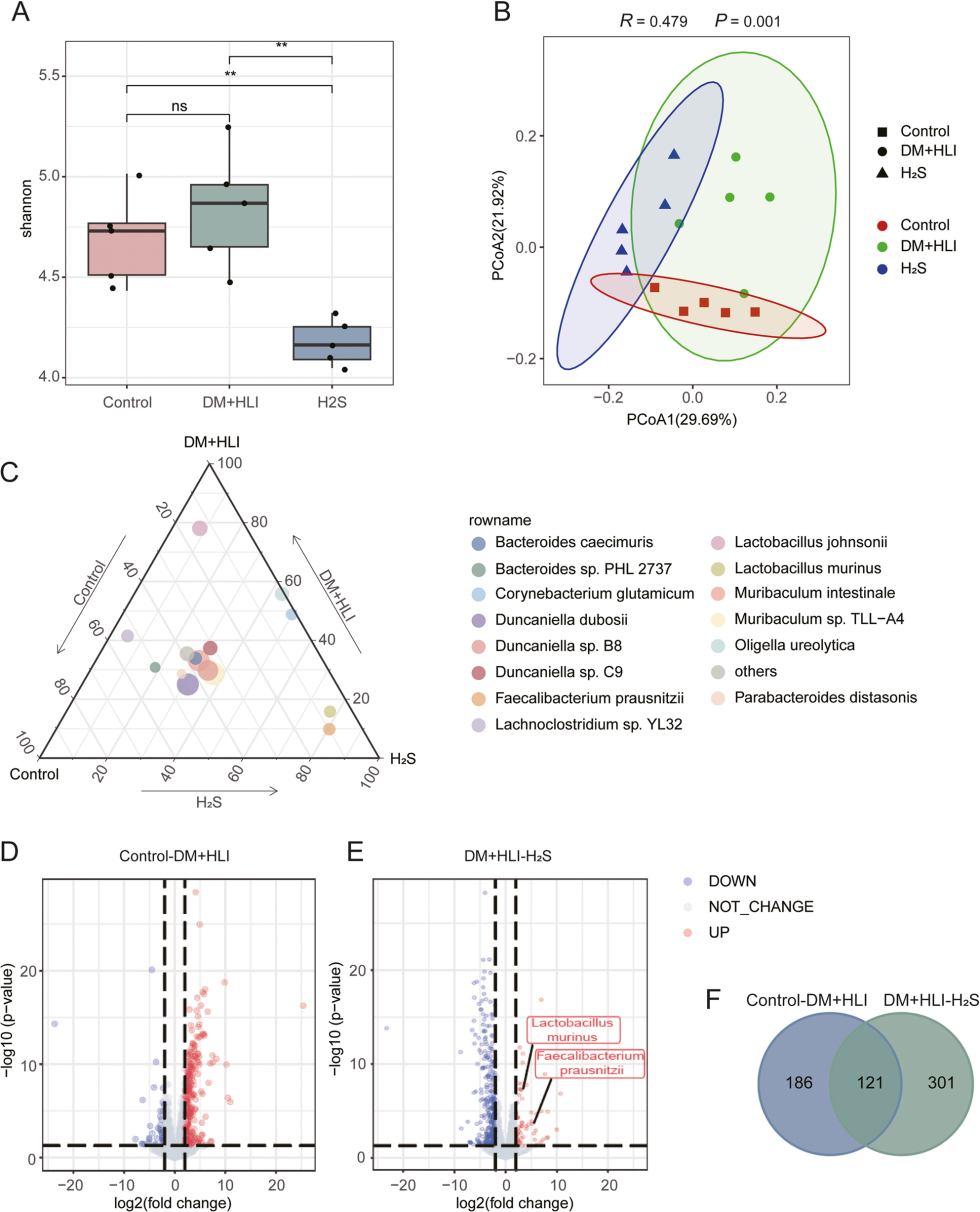
Species composition of the fourth sample collection and DESeq2 analysis of taxonomy for the differences. **A** Shannon index for the α diversity. **B** PCoA for the β diversity. **C** Ternary plot illustrating species-level compositional differences across groups. Each vertex represents a group; symbol size corresponds to relative abundance; each color represents a distinct species. **D** Volcano plots for the differences between control group and DM + HLI group at the species level (*P* < 0.05, log_2_FoldChange > 2). **E** Volcano plots for the differences between DM + HLI group and H_2_S group at the species level (*P* < 0.05, log_2_FoldChange > 2). **F** Venn diagram depicting the number of shared and unique differentially abundant bacterial species across comparisons. Data are presented as mean ± SEM (*n* = 5; ^ns^ *P* > 0.05, ^**^ *P* < 0.01 by Wilcoxon test with Benjamini-Hochberg FDR adjustment applied specifically to pairwise comparisons among multiple groups). DM, diabetes mellitus; HLI, hindlimb ischemia; H_2_S, Hydrogen Sulfide; PCoA, principal coordinates analysis; DESeq2, Differential Expression analysis of Sequencing data version 2; SEM, standard error of the mean; FDR, false discovery rate

To effectively demonstrate the impact of H_2_S on the gut microbiota in mice with diabetes and HLI, a thorough analysis of the fourth fecal sample results was conducted. The species composition of intestinal microbiota among the three groups exhibits significant differences in both α and β diversity, and H_2_S intervention could substantially alter the abundance of various microbial communities (Fig. 4A, B). The ternary plot shows the top 15 species by total abundance. Notably, H_2_S significantly increased the abundance of *Lactobacillus murinus* and *Faecalibacterium prausnitzii* (Fig. 4C). The volcano plot generated from the DESeq2 analysis revealed the microbial biomarkers for the three groups. In the DM group, 39 species were down-regulated, and 268 were up-regulated compared with the control group. In the H_2_S group, 359 species were down-regulated and 63 species (including *Lactobacillus murinus* and *Faecalibacterium prausnitzii*) were up-regulated compared to the DM group. One hundred twenty-one microbial biomarkers showed significant differences, as indicated by overlap between the two DESeq2 analyses (Fig. 4D-F).

**Fig. 5:**
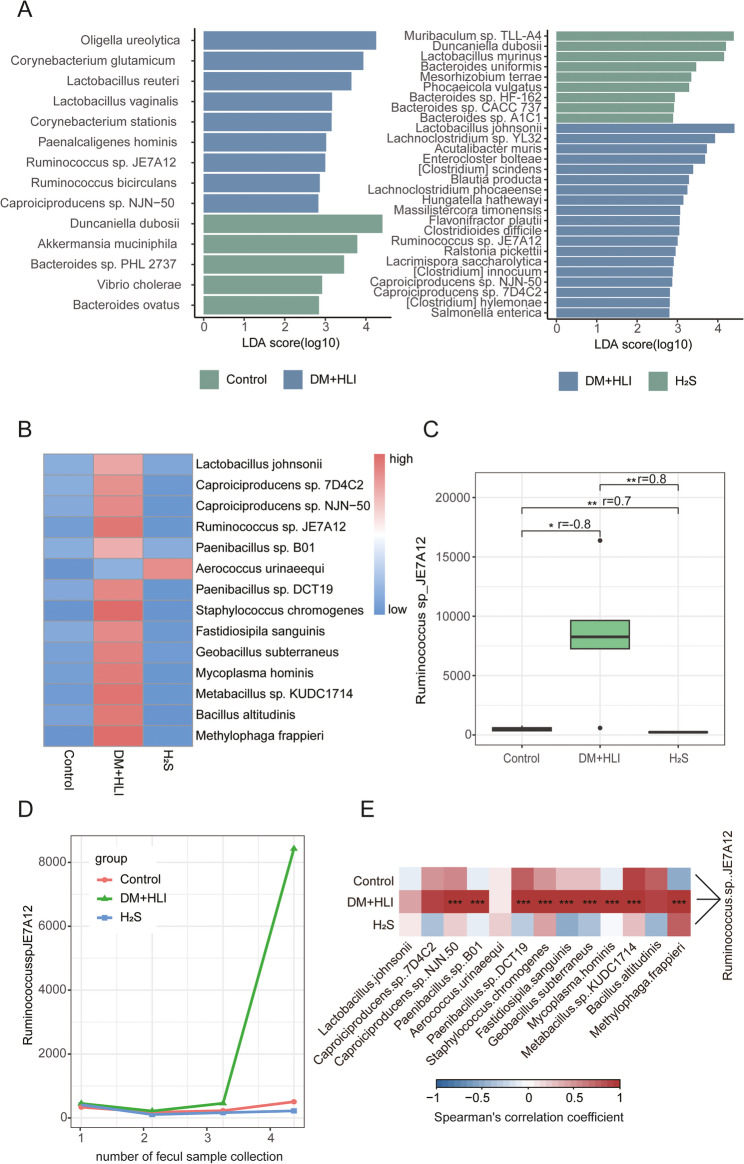
Differential abundance analysis using LEfSe and the correlation between different bacteria. **A** Histogram for LDA value (LDA > 2.8). **B** Heatmap for 14 species with differential abundance among the three groups. The color bar represented the relative abundance of the species (red indicates high abundance, blue indicates low abundance). **C** The abundance difference of *Ruminococcus sp. JE7A12* among the three groups at the fourth sample collection. **D** Abundance changes of *Ruminococcus sp. JE7A12* during four sample collections. **E** *Spearman* correlation analysis with Benjamini-Hochberg FDR correction between *Ruminococcus sp. JE7A12* and 13 other differentially abundant bacterial species. Data are presented as mean ± SEM (*n* = 5; ^*^ *P* < 0.05, ^**^P < 0.01, ^***^ *P* < 0.001 by Wilcoxon test with Benjamini-Hochberg FDR adjustment applied specifically to pairwise comparisons among multiple groups). Effect sizes were quantified using the correlation coefficient *r* (calculated as *r* = *Z* / √*N*, where *Z* is the test statistic and *N* is the total sample size). DM, diabetes mellitus; HLI, hindlimb ischemia; H_2_S, Hydrogen Sulfide; LDA, Linear Discriminant Analysis; LefSe, Linear Discriminant Analysis Effect Size; SEM, standard error of the mean; FDR, false discovery rate

LDA (lefse) analysis also examined the intergroup differences in the composition of the gut microbiota. When the LDA value was set to 2.8, nine species of intestinal microbiota were found to be more abundant in the DM + HLI group than in the control group. Similarly, the H_2_S group showed higher abundances of 9 intestinal microbiota species than the DM + HLI group (Fig. 5A). To investigate the effect of H_2_S intervention on the gut microbiota in diabetic mice with HLI, the results of DESeq2 and LDA analyses were intersected to enhance the representativeness of the findings. Fourteen species displayed significant differences among the three groups (Fig. 5B). One species that stood out to us is *Ruminococcus sp. JE7A12*, a member of the *Ruminococcus* genus. The difference of *Ruminococcus sp. JE7A12* abundance among the three groups was shown in Fig. 5C. Notably, the abundance of *Ruminococcus sp. JE7A12* in the DM + HLI group was significantly higher than that in the other two groups. For these comparisons, the values of |*r*| exceeded 0.5, indicating large effect sizes. The dynamic change of *Ruminococcus sp. JE7A12* abundance was presented in Fig. 5D. The increase of *Ruminococcus sp. JE7A12* abundance primarily occurred after HLI surgery, and H_2_S could significantly mitigate these changes (Fig. 5D). Additionally, a correlation analysis between *Ruminococcus sp. JE7A12* and 13 other differential bacteria was conducted. The abundance of *Ruminococcus sp. JE7A12* was positively correlated with 9 of these bacterial species in the DM + HLI group (Fig. 5E). Linear regression analyses showed that *Ruminococcus sp. JE7A12* and the 9 species above showed significant negative correlations with the blood perfusion ratio (Fig. S4).

**Fig. 6:**
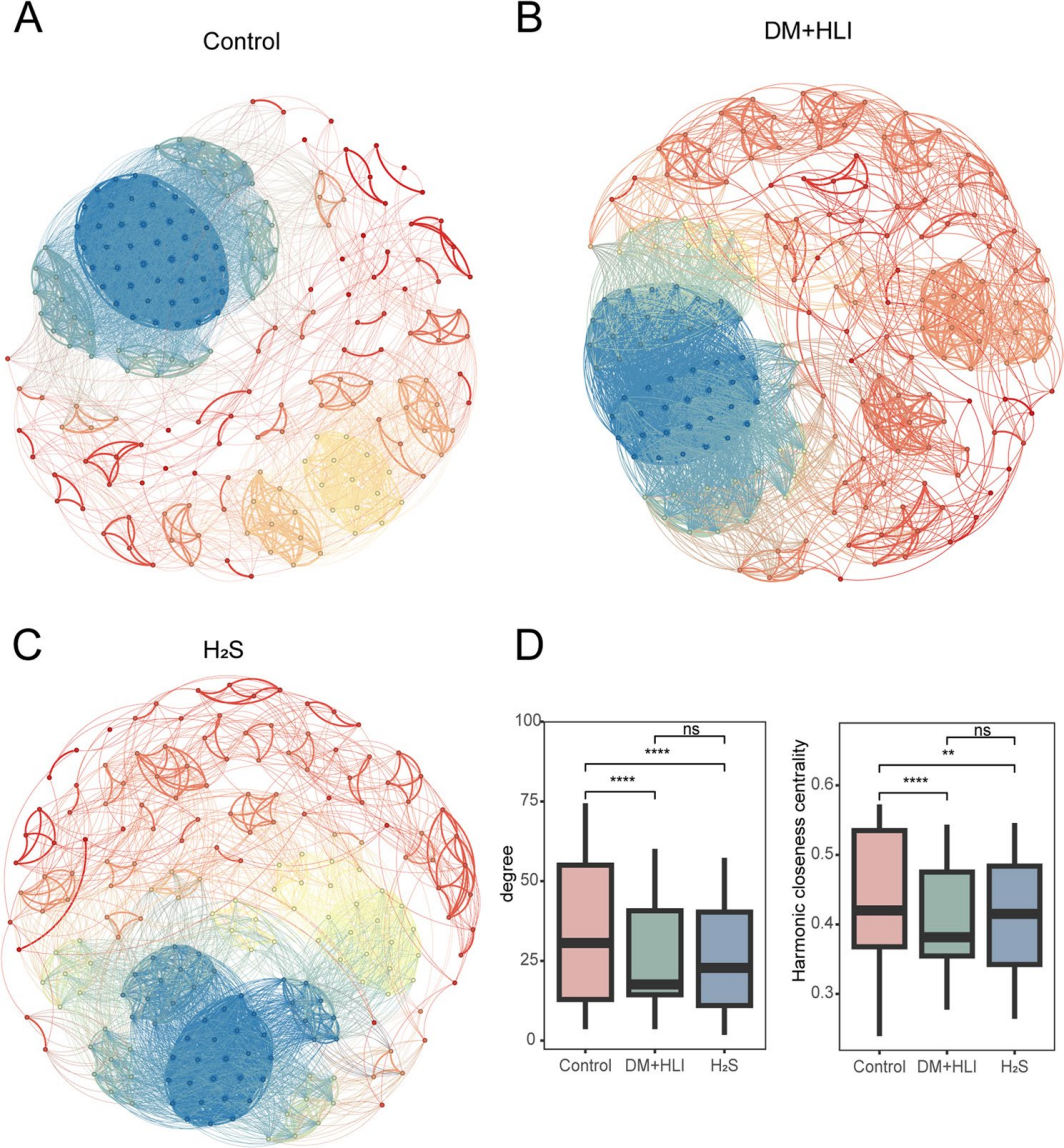
Comparative analysis of gut microbiome network characteristics. **A**-**C** Network graphs depicting the co-occurrence patterns among microbial communities. The nodes represented distinct species and edges indicated significant co-occurrence relationships. **D** Box charts for key network features. The degree represented the number of connections of the nodes, and the harmonic closeness centrality indicated the average distance between nodes. Data are presented as mean ± SEM (*n* = 5; ^ns^ *P* > 0.05, ^**^ *P* < 0.01, ^****^ *P* < 0.0001 by Wilcoxon test with the Benjamini-Hochberg FDR adjustment applied specifically to pairwise comparisons among multiple groups). DM, diabetes mellitus; HLI, hindlimb ischemia; H_2_S, Hydrogen Sulfide; SEM, standard error of the mean; FDR, false discovery rate

Co-occurrence networks were constructed using the top 200 abundant species across the three groups to characterize microbial interactions (Fig. 6A-C). Microbial network analysis revealed that the control group had greater complexity than the DM + HLI and H_2_S groups, with no statistically significant difference between the DM + HLI and H_2_S groups (Fig. 6A-C). The degree of nodes and harmonic closeness centrality were significantly higher in the control group than in the DM + HLI and H_2_S groups (Fig. 6D).

### Comprehensive functional profiling using KEGG pathway analysis

**Fig. 7:**
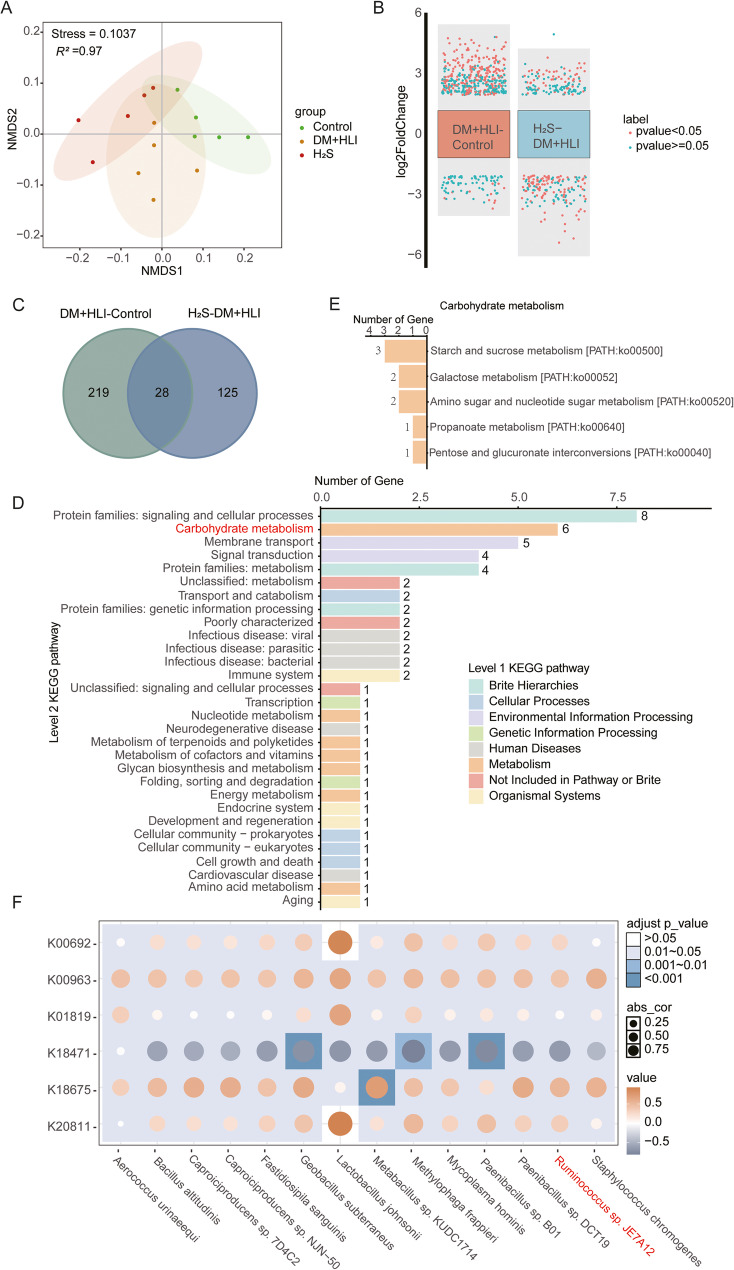
Analysis of gene composition and functional profiles across groups. **A** NMDS analysis. **B** Volcano plots identifying differentially abundant genes. Red points denote genes with significant differences (*P* < 0.05, log_2_FoldChange > 2), and blue points represent non-significant genes. **C** Venn diagram illustrating the number of unique and shared genes among groups. **D**, **E** KEGG pathway enrichment analysis of the differentially abundant genes. **F**) *Spearman* correlation matrix between 6 key differentially abundant genes and 14 differential microbial species. The size of each circle corresponds to the absolute value of the correlation coefficient, and the color indicates the direction (positive or negative). The color intensity of the square block represents the *p*-value of the correlation, with darker colors corresponding to smaller (more significant) *p*-values. Notably, *Ruminococcus sp. JE7A12* showed significant positive correlations with four genes (K00692, K00963, K18675, and K20811). The resulting *p-values* were adjusted for multiple comparisons using the Benjamini-Hochberg FDR method to control for type I errors. (*n* = 5). DM, diabetes mellitus; HLI, hindlimb ischemia; H_2_S, Hydrogen Sulfide; NMDS, non-metric multidimensional scaling; KEGG, Kyoto Encyclopedia of Genes and Genomes; FDR, false discovery rate

NMDS of metagenomic data projected the genetic composition into two-dimensional space, revealing distinct clustering patterns among the three experimental groups (Fig. 7A). Differential gene expression analysis using DESeq2 identified 235 significantly upregulated and 12 downregulated genes in the DM + HLI group compared to controls (Fig. 7B, C). When comparing the H_2_S treatment group to the DM + HLI group, we detected 55 upregulated and 98 downregulated genes (Fig. 7B, C). Functional metagenomic profiling identified 28 differentially expressed microbial genes, which were subsequently mapped to KEGG pathways for functional annotation. These genes mapped to 8 level 1 and 30 level 2 KEGG pathways (Fig. 7D), with 6 genes particularly enriched in carbohydrate metabolism pathways (Fig. 7E). The associated level 3 pathways included starch and sucrose metabolism, galactose metabolism, amino sugar and nucleotide sugar metabolism, propanoate metabolism, and pentose and glucuronate interconversions (Fig. 7E). Correlation analysis was conducted between the 14 differentially abundant bacterial species and the 6 carbohydrate metabolism-related genes (Fig. 7F). Notably, *Ruminococcus sp. JE7A12* was significantly positively correlated with four genes (K00692, K00963, K18675, K20811), raising the possibility that this bacterium harbors these genes. In contrast, it was negatively correlated with K18471 and showed no significant correlation with K01819 (Fig. 7F).

## Discussion

Diabetes is a significant risk factor for HLI, with patients who have both diabetes and PAD facing a fivefold increased risk of amputation compared to those without diabetes [26]. However, current pharmacological treatments for PAD primarily focus on preventing acute arterial embolism, offering limited strategies to reduce limb damage and improve lower limb function. Consequently, there is an urgent need for comprehensive research and exploration in the prevention and treatment of PAD in diabetic patients [27]. This study demonstrates that H_2_S could improve blood perfusion in diabetic mice with HLI and protect the structure and function of ischemic skeletal muscle. Furthermore, H_2_S could modulate the distribution of gut microbiota and maintain the balance of the intestinal microecology.

Previous research has demonstrated that diabetes reduces H_2_S production in patients and in mice [18]. A similar trend was observed in our study. A reduction in H_2_S levels may lead to decreased insulin secretion and diminished insulin sensitivity, both of which contribute to the pathogenesis of diabetes [28]. Therefore, the decrease in H_2_S levels and the worsening of diabetes could potentially create a vicious cycle. Previous studies have also demonstrated that exogenous administration of H_2_S could enhance GLP-1 secretion and improve oral glucose tolerance in obese mice under stress [29]. In the db/db mice, intraperitoneal injections of NaHS for 16 weeks could alleviate FBG [30]. However, the 21-day H_2_S treatment in this study did not result in a significant improvement in the mice’s blood glucose levels. We propose that the differences observed above could be due to variations in the diabetes models employed.

Diabetes could decrease H_2_S production in vascular endothelial cells and vascular smooth muscle cells by inhibiting the expression of cystathionine-lyase (CSE) and 3-mercaptopyruvate sulfurtransferase [18, 31, 32]. This reduction results in elevated levels of anti-angiogenic factors and decreased levels of pro-angiogenic factors [32]. By inhibiting the activation of the NOD-like receptor pyrin domain-containing three inflammasome or by regulating endoplasmic reticulum stress, H_2_S could delay the progression of diabetes and its chronic complications, such as diabetes-accelerated atherosclerosis [33, 34]. Previous studies by our team demonstrated that lower H_2_S production in the vascular smooth muscle cells of diabetic rats was involved in the development of macrovascular complications associated with diabetes by enhancing autophagic activity [18]. Findings have also indicated that the use of H_2_S donors or the overexpression of CSE could significantly improve blood perfusion in the lower limbs of diabetic mice after experiencing ischemia [31]. In this study, H_2_S administration significantly enhanced blood perfusion in the lower limbs of diabetic mice with HLI. Meanwhile, the current research demonstrated that H_2_S intervention preserved the structural integrity and function of skeletal muscle in the lower limbs of diabetic mice with HLI, which aligns with previous findings [35, 36]. A comprehensive investigation into the mechanism by which H_2_S enhances blood perfusion in the lower limbs could provide a new therapeutic target for the treatment of diabetic lower limb ischemia.

The gut microbiota contributes to the onset of diabetes by impairing glucose tolerance and aggravating insulin resistance [6, 37]. This is probably linked to the low-grade inflammatory response induced by gut microbiota dysbiosis [38]. An imbalance in gut microbiota has also been observed in multiple models of chronic diabetic complications, including diabetic nephropathy, diabetic retinopathy, coronary artery disease, and cerebrovascular diseases [37, 39]. Lin Zhu and his colleagues [40] discovered gut microbiota dysbiosis in ischemic mice. We observed a similar phenomenon in our experiment, identifying an imbalance in the gut microbiota of diabetic mice with HLI. *Bacteroidetes* and *Firmicutes* are among the most dominant bacterial groups in the gut microbiome [41]. With advancing age, the *Firmicutes*-to-*Bacteroidetes* ratio tends to increase, and this ratio may also vary in the presence of certain disease [42]. Patients with type 2 diabetes or obesity exhibited a higher *Firmicutes*-to-*Bacteroidetes* ratio in the gut microbiota, which was associated with the expression of inflammatory mediators [43, 44]. In these animal experiments, we observed a higher *Firmicutes*-to-*Bacteroidetes* ratio in diabetic mice with HLI than in the control group. Notably, following successful diabetes induction with STZ, the *Firmicutes*-to-*Bacteroidetes* ratio in the DM + HLI and H_2_S groups did not differ significantly from that in the control group. This finding implies that STZ administration itself has minimal immediate impact on this ratio, and the early hyperglycemic phase (within three days post-modeling) similarly demonstrates negligible effects. Our results are consistent with reports by Jenna I. Wurster et al. [45]. Nevertheless, the existing literature indicates that STZ-induced diabetic models exhibit significant alterations in gut microbial diversity, composition, and abundance over extended periods (several weeks) [46]. The temporal dynamics of STZ and hyperglycemia effects on gut microbiota, notably the transition from immediate to delayed impacts, warrant further systematic investigation. In addition, the equilibrium of gut microbial communities is shaped not only by their composition and abundance but also by microbial interactions. Gut microbial communities with higher interaction complexity and greater taxonomic diversity typically exhibit enhanced ecosystem stability [47]. In this study, the complexity of gut microbial communities was significantly reduced by the DM + HLI model. In subsequent research, we aim to investigate further the mechanisms by which an imbalance in gut microbiota facilitates the progression of lower limb ischemia.

As a gaseous signaling molecule, H_2_S plays a pivotal role in various pathophysiological processes and significantly regulates the gut microbiota [11, 12]. Its anti-inflammatory effects operate through multi-level mechanisms: at the systemic level, H_2_S exhibits dose-dependent regulation, simultaneously inhibiting the Nuclear Factor kappa-light-chain-enhancer of activated B cells (NF-κB) pathway and the NLR Family Pyrin Domain Containing 3 (NLRP3) inflammasome [48] while activating the Nuclear Factor Erythroid 2-Related Factor 2 (Nrf2) pathway [49], significantly reducing the expression of inflammatory cytokines such as IL-6 and IL-1β [48, 49]. This study confirmed that H_2_S intervention lowered plasma levels of inflammatory cytokines in DM + HLI mice. At the intestinal microecological level, both endogenous and exogenous H_2_S at a physiological range not only maintains microbial membrane stability and inhibits the production of pathogenicity factors [50], but also reshapes gut microbiota balance by enhancing anti-inflammatory bacterial abundance while suppressing pro-inflammatory bacteria. Our experiments identified two specifically enhanced beneficial bacteria: *Faecalibacterium prausnitzii*, an anti-inflammatory bacterium crucially involved in diabetes pathogenesis [51] whose depletion correlates with inflammatory diseases [52] and diabetes [53]; and *Lactobacillus murinus*, which is closely associated with glycerophospholipid metabolism, sphingolipid metabolism, and aromatic amino acid biosynthesis pathways [54] and can be elevated by certain anti-diabetic medications [55]. Conversely, H_2_S effectively suppressed pro-inflammatory *Ruminococcus species*, particularly *Ruminococcus sp. JE7A12*, whose elevation is characteristic of gut dysbiosis [56]. This genus exhibits potent mucin-degrading activity that impairs intestinal barrier function and drives immune dysregulation [57]. Its abundance is notably elevated in patients with type 2 diabetes [53], and *Ruminococcus gnavus* specifically shows a positive correlation with T2D prevalence [58]. We propose that H_2_S modulates the critical balance between inflammatory and anti-inflammatory bacterial populations in diabetic HLI mice. Consistent with this, the attenuation of inflammation may improve vascular endothelial function and promote angiogenesis in ischemic tissues, representing a potential key mechanism for the observed improvement in perfusion [32]. At the muscular level, H_2_S-mediated remodeling of the gut microbiota may facilitate tissue repair through the restoration of beneficial microbial metabolites. The emerging “gut–muscle axis” concept posits that gut microbiota-derived metabolites serve as key signaling molecules linking intestinal health to skeletal muscle physiology. Dysbiosis marked by a high *Firmicutes*-to-*Bacteroidetes* ratio and enrichment of pro-inflammatory bacteria is often linked to reduced levels of beneficial metabolites such as SCFAs [59, 60]. SCFAs are known to modulate lipid, carbohydrate, and protein metabolism in skeletal muscle and may act as important regulators of muscle metabolism and function [61]. Beyond SCFAs, other microbiota-derived metabolites such as bile acids and tryptophan derivatives have also been implicated in muscle homeostasis through their effects on mitochondrial function, lipid and glucose metabolism, and inflammation [62, 63]. Thus, the H_2_S-induced shift toward a more balanced gut microbiota may enhance the production of SCFAs and other protective metabolites, thereby improving skeletal muscle metabolism and contractility. This offers a plausible mechanistic explanation for the muscle structure and functional recovery observed in H_2_S-treated mice [35, 36]. Supporting this notion, through the catabolism of various carbon sources by fecal microbiota, metabolites including SCFAs are generated [64]; consistent with this, we observed that 6 differentially expressed genes were enriched in carbohydrate metabolism pathways, including starch and sucrose metabolism and propanoate metabolism, which are closely related to SCFA production. However, direct measurements of microbial metabolites (e.g., SCFAs, bile acids, tryptophan derivatives) in plasma or fecal samples were not performed in this study. Future investigations incorporating targeted metabolomic profiling are warranted to identify which specific metabolites are altered by H_2_S treatment and to generate hypotheses regarding their potential roles in mediating the observed effects. Additionally, whether H_2_S exerts direct effects on the growth of key bacterial species identified in this study (e.g., *Ruminococcus sp. JE7A12*, *Lactobacillus murinus*, *Faecalibacterium prausnitzii*) remains to be determined. In vitro experiments examining bacterial growth kinetics under H_2_S treatment would help clarify direct versus indirect mechanisms. These lines of investigation will be essential to fully elucidate how H_2_S modulates the gut–muscle axis through microbial metabolites and to validate the causal role of specific bacterial taxa in mediating vascular and muscular recovery.

Prior research has established H_2_S-gut microbiota crosstalk in metabolic diseases (e.g., metabolic syndrome, MASLD/MASH) [11, 13, 15–17] and vascular-protective effects of H_2_S [18, 19], yet these aspects have remained largely separate. Using a diabetic HLI model, we integrated these findings by demonstrating that H_2_S concomitantly improves vascular/muscular function and induces a beneficial microbial shift. We propose that this targeted microbiota remodeling, associated with functional recovery, defines a novel therapeutic mechanism for H_2_S in vascular complications.

Compared to conventional microbiota-targeting approaches, H_2_S intervention exhibits a fundamentally distinct mechanism of action. While fecal microbiota transplantation aims to reconstruct the gut microbiota by directly transferring donor fecal material, it faces challenges, including variable safety profiles, donor dependency, and standardization issues [65]. Similarly, probiotic therapies for diabetes and its complications primarily rely on the introduction of specific exogenous bacterial strains [37]. In contrast, H_2_S appears to modulate the endogenous microbial ecosystem by selectively enhancing autochthonous beneficial bacteria, such as *Faecalibacterium prausnitzii*, while suppressing inflammation-associated species, such as *Ruminococcus sp. JE7A12*. This endogenous modulation strategy may offer distinct advantages for maintaining ecological balance and avoiding colonization resistance, which is often encountered with exogenous microbial interventions. Furthermore, unlike single-target therapies focused solely on microbial composition, H_2_S has the potential to concurrently address both vascular dysfunction and microbial dysbiosis, representing a uniquely synergistic therapeutic strategy for diabetic vascular complications.

Based on our findings, we propose a translational roadmap for developing H_2_S-based therapies. The preclinical phase requires optimizing delivery routes, validating safety profiles, and confirming key biomarkers: including specific bacterial taxa and inflammatory markers. Potential synergies with existing PAD treatments should also be investigated. In clinical research, the initial focus should be on diabetic PAD patients with gut microbiota dysbiosis patterns similar to those in our model. Controlled clinical trials are needed to evaluate safety and therapeutic potential, while microbiota-based biomarkers may enhance patient stratification and treatment monitoring. Our current findings are still at a preliminary stage, and we look forward to further in-depth investigations that may translate into clinically meaningful therapeutic or diagnostic pathways.

### Limitations and future directions

This study has several limitations. Firstly, and most importantly, a major limitation of this study is the absence of a sham-operated control group (anesthesia + skin incision without femoral artery ligation). As the surgical procedure itself may induce stress and potentially influence gut microbiota, the specific effects attributable solely to HLI versus surgical stress cannot be fully deconvoluted in our experimental design. Furthermore, the isolated impact of surgical stress alone on the murine gut microbiota remains incompletely characterized. Therefore, future studies should include a sham-operated control group to better disentangle the confounding influence of surgical stress from the pathophysiological consequences of HLI. Secondly, at the time of initial fecal sampling, differences in the abundance of *Firmicutes* and *Bacteroidetes* were observed among the groups, which may have introduced confounding factors during the cage-housing process. Fortunately, these intergroup differences were absent in the fecal samples collected during the second sampling (three days after STZ modeling), ensuring the comparability of the subsequent experimental groups. Thirdly, this study did not examine the molecular mechanisms by which H_2_S modulates the gut microbiota to improve lower limb blood flow in diabetic mice with HLI. Fourthly, the sample size in this study, though consistent with numerous previous metagenomic analyses investigating gut microbiota in mouse models of disease, may limit the statistical power to detect significant changes in rare taxa. Fifthly, this study did not include qPCR or targeted sequencing validation of microbial gene expression changes, nor metabolomic analysis to confirm functional shifts. Future research integrating these approaches is needed to confirm gene alterations and link them to metabolic functions, reinforcing the mechanistic role of H_2_S-induced microbial shifts. Sixthly, only male mice were used in this study. While this follows standard practice for diabetes and HLI models, gut microbiota and H₂S signaling can differ by sex. Thus, our findings may not be fully generalizable to females, and future studies should include both sexes. Finally, although this study demonstrates clear associations between H_2_S therapy, gut microbiota remodeling, and functional recovery, causality remains to be established. To address this, future studies must test whether (1) fecal microbiota transplantation from H₂S-treated donors recapitulates the phenotype, (2) depletion of *Ruminococcus sp. JE7A12* improves outcomes, and (3) mono-colonization with *Lactobacillus murinus* or *Faecalibacterium prausnitzii* is sufficient to confer benefit. Such mechanistic investigations will be essential to definitively link H_2_S, gut microbiota modulation, and improved outcomes in diabetic vascular complications. Future research should also focus on in-depth analysis of relevant microbial metabolites (e.g., short-chain fatty acids, bile acids) and H_2_S-producing bacteria, with an emphasis on elucidating the underlying molecular mechanisms.

## Conclusions

**Fig. 8:**
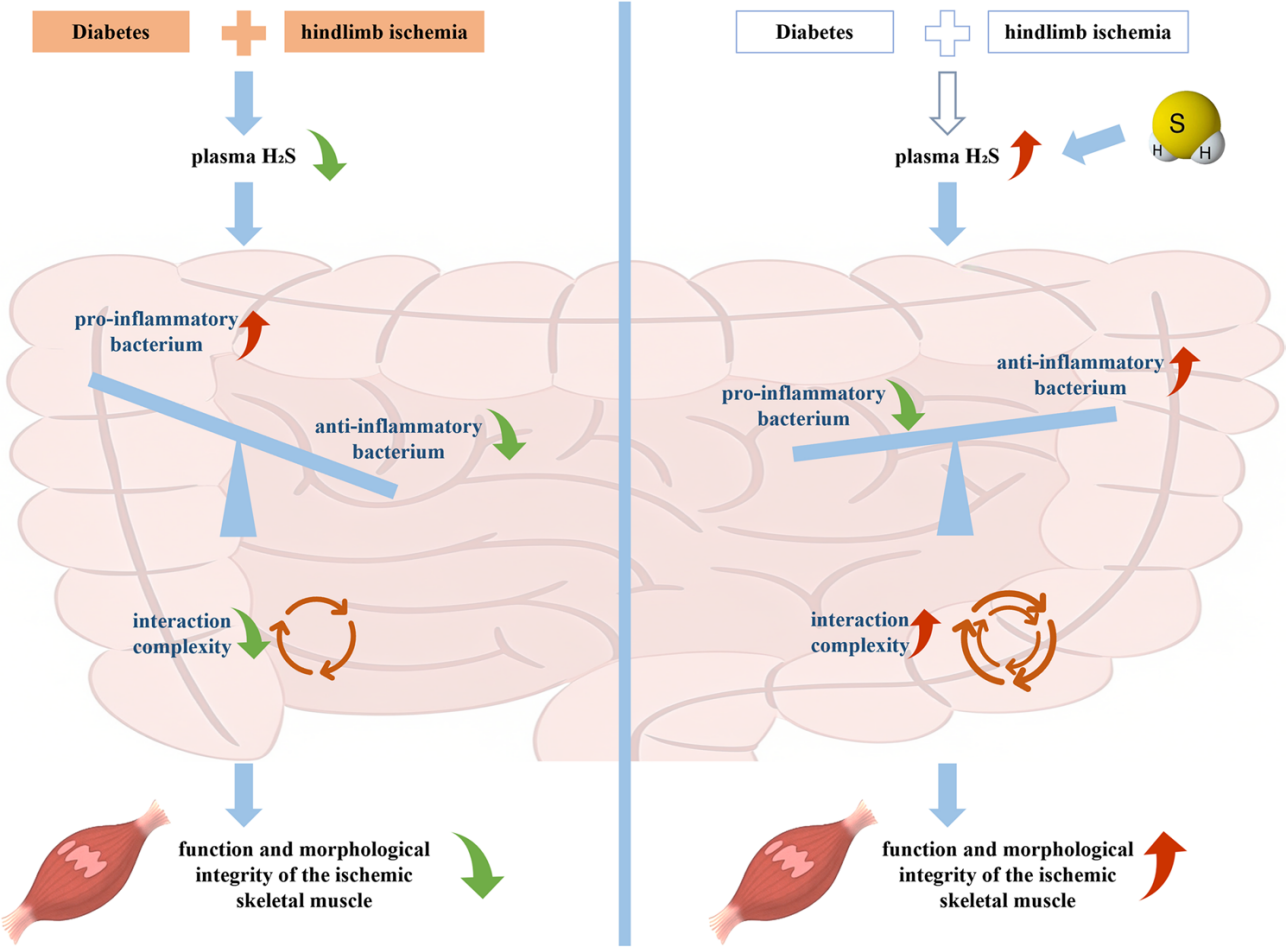
Summary of the effects of H_2_S on gut microbiota and ischemic recovery in diabetic HLI mice. H_2_S, hydrogen sulfide

The DM + HLI model induces gut microbiota dysbiosis in mice. H_2_S could promote the recovery of lower limb blood flow and ameliorate structural and functional damage in the ischemic gastrocnemius muscle, in diabetic mice with HLI. Concurrently, exogenous H_2_S administration could alter the abundance of intestinal microbiota, thereby improving microbial balance in diabetic mice with HLI (Fig. 8). These findings suggest that the therapeutic benefits of H_2_S in diabetic HLI may involve direct vascular effects and modulation of the gut microbiota. However, the exact causal relationship requires further validation. Future studies will focus on elucidating the mechanistic interplay between gut microbiota and skeletal muscle pathophysiology to unravel how H_2_S improves peripheral vascular and muscular recovery fully.

## Supplementary Information

Supplementary Material 1.

Supplementary Material 2.

Supplementary Material 3.

Supplementary Material 4.

Supplementary Material 5.

## Data Availability

The raw sequence data reported in this paper have been deposited in the Genome Sequence Archive (Genomics, Proteomics & Bioinformatics 2021) in National Genomics Data Center (Nucleic Acids Res 2022), China National Center for Bioinformation / Beijing Institute of Genomics, Chinese Academy of Sciences (GSA: CRA027017) that are publicly accessible at https://ngdc.cncb.ac.cn/gsa/browse/CRA027017.

## Abbreviations

CSE: Cystathionine-lyase
DESeq2: Differential Expression analysis of Sequencing data version 2
DM: Diabetes mellitus
FBG: Fasting blood glucose
GLP-1: Glucagon-Like Peptide-1
H_2_S: Hydrogen Sulfide
H&E: Hematoxylin and Eosin
HLI: Hindlimb Ischemia
IL-1β: Interleukin-1 β
IL-6: Interleukin-6
KEGG: Kyoto Encyclopedia of Genes and Genomes
LDA: Linear Discriminant Analysis
LEfSe: Linear Discriminant Analysis Effect Size
NaHS: Sodium Hydrosulfide
NMDS: Non-Metric Multidimensional Scaling
PAD: Peripheral Artery Disease
PCoA: Principal Coordinates Analysis
STZ: Streptozotocin
TEM: Transmission Electron Microscopy
