# Supplementary material for: The impact of hydrogen sulfide on gut microbiota of diabetic mice with lower limb arterial ischemia
Source: BMC Microbiol. 2026 Jun 5;26:729. doi: 10.1186/s12866-026-05167-5 (PMC13474665; doi:10.1186/s12866-026-05167-5)
Supplement: Supplementary file 1 [file 12866_2026_5167_MOESM5_ESM.docx]

**Data Analysis**

Sequencing adapters from the raw reads were removed using Cutadapt. Low-quality reads were trimmed using a sliding window algorithm implemented in fastp. Sequences originating from mice were filtered out with Bowtie2, using the mouse reference genome (mm39) (https://www.ncbi.nlm.nih.gov/datasets/genome/GCF_000001635.27/). Taxonomic classification of the high-quality reads was performed using Kraken2, against the Mouse Gastrointestinal Bacteria Catalogue (MGBC) database. Relative abundance was then normalized using Bracken. Subsequently, Megahit was used to assemble contigs from high-quality reads, and Prodigal was employed to predict coding sequences (CDSs). Redundant predicted genes were removed using CD-HIT. Gene read coverage across different samples was estimated using Salmon. Finally, gene functional annotation was performed using eggNOG-mapper. The resulting tables of taxonomic classification and gene annotations were then used for further analysis.
